# Efficacy and Safety of MITSU AB™ Triclosan-Coated Sutures in Preventing Surgical Site Infections

**DOI:** 10.7759/cureus.84246

**Published:** 2025-05-16

**Authors:** Pushkar Galam, Yogesh Desai, Army Patel, Lalit Bajpayee, Kiran Kumar Shetty

**Affiliations:** 1 Surgery, Hope Hospital, Pune, IND; 2 Obstetrics and Gynecology, Shanti Hospital, Udvada, IND; 3 Product Performance and Engineering, Meril Life Sciences, Vapi, IND

**Keywords:** absorbable suture, antimicrobial resistance, surgical site infection, triclosan-coated sutures, wound healing

## Abstract

Introduction: Surgical site infections (SSIs) are a significant source of postoperative morbidity. Triclosan-coated absorbable sutures, such as MITSU AB™ (Meril Endo Surgery Private Limited, Vapi, India), are designed to inhibit early microbial colonization, reducing the risk of SSIs. However, concerns about triclosan resistance highlight the need for real-world clinical evaluation. The present study aims to assess wound healing outcomes, incidence of SSIs, and triclosan resistance in patients treated with MITSU AB™ absorbable triclosan-coated sutures.

Methods: A prospective, single-arm, multi-center post-marketing surveillance study was conducted involving 60 patients undergoing general surgical procedures, including laparoscopic appendectomy, episiotomy, and soft tissue repair. Patients were monitored postoperatively at discharge and days 7, 15, and 30. Primary outcomes included SSI occurrence and triclosan resistance. Secondary endpoints were wound healing grade, pain (measured by visual analogue scale (VAS)), and suture handling feedback.

Results: The study population had a mean age of 38.5 ± 17.4 years, with 36 (60%) female participants. All patients (100%) achieved complete wound healing (Grade A) by day 30. No SSIs, triclosan-resistant microbial growth, or adverse events were recorded. Pain scores decreased over time, with 59 (98.3%) patients pain-free by day 15 and 100% by day 30. Suture handling was rated excellent in 51 (85%) cases. No re-interventions or re-admissions due to infection occurred during the study.

Conclusion: MITSU AB™ triclosan-coated sutures demonstrated excellent infection prevention, complete healing, and favorable handling with no evidence of triclosan resistance. These findings support their safe and effective use in clinical surgery.

## Introduction

Absorbable sutures are critical in surgical wound closure, especially for internal tissues where removal is impractical. However, these materials are susceptible to microbial colonization, potentially leading to surgical site infections (SSIs), one of the most common postoperative complications worldwide. To mitigate this risk, absorbable sutures coated with the antimicrobial agent triclosan have been developed to provide localized infection control during the early, high-risk phase of wound healing [1,2].

Triclosan acts as a broad-spectrum antimicrobial agent by inhibiting the bacterial enzyme enoyl-acyl carrier protein reductase (FabI), a critical component of the fatty acid biosynthesis pathway. This mechanism disrupts bacterial cell membrane formation, leading to growth inhibition or cell death depending on concentration [3]. Triclosan-coated absorbable sutures such as polyglactin 910 (Vicryl Plus) and poliglecaprone 25 (Monocryl Plus) have shown strong in vitro and in vivo antibacterial activity against common pathogens, including *Staphylococcus aureus*, *Staphylococcus epidermidis*, *Escherichia coli*, and their drug-resistant variants [4,5]. These sutures create a zone of inhibition that prevents early microbial colonization during the first days post-surgery, when tissues are most vulnerable [6].

The introduction of products like MITSU AB™ absorbable triclosan-coated sutures (Meril Endo Surgery Private Limited, Vapi, India) represents an effort to harness this antimicrobial benefit while maintaining absorbability and tensile integrity. Yet, despite widespread use, there are growing concerns about bacterial adaptation and potential resistance to triclosan, particularly as it has been used extensively in both medical and consumer products [7]. Some studies suggest that triclosan's effectiveness may be limited against certain Gram-negative organisms or biofilm-forming strains in vivo, depending on suture type, coating uniformity, and environmental factors [8,9].

Given the critical role of absorbable sutures in reducing postoperative infections and supporting tissue healing, this study explores the antimicrobial performance and resistance patterns associated with MITSU AB™ absorbable triclosan-coated sutures. By focusing on colonization behavior and potential resistance in clinical pathogens, this research aims to evaluate the continued utility and limitations of triclosan-coated absorbable materials in surgical practice.

## Materials and methods

Study design

This was a prospective, single-arm, multi-center, observational study designed to evaluate triclosan-resistant pathogenic colonization in patients treated with MITSU AB™ absorbable poly(glycolide/L-lactide) triclosan-coated surgical sutures following soft tissue approximation. Conducted in real-world clinical settings across two sites in India, namely, Hope Hospital in Pune and Shanti Hospital in Udvada, the study enrolled a total of 60 subjects who underwent a range of general surgical procedures, including laparoscopic appendectomies, episiotomies, and minor trauma repairs. Subjects were followed up for a 30-day postoperative period, with clinical assessments scheduled at discharge and days 7, 15, and 30. Microbial cultures, wound healing evaluations, and pain assessments were systematically performed during each follow-up.

Study device

**Figure 1:**
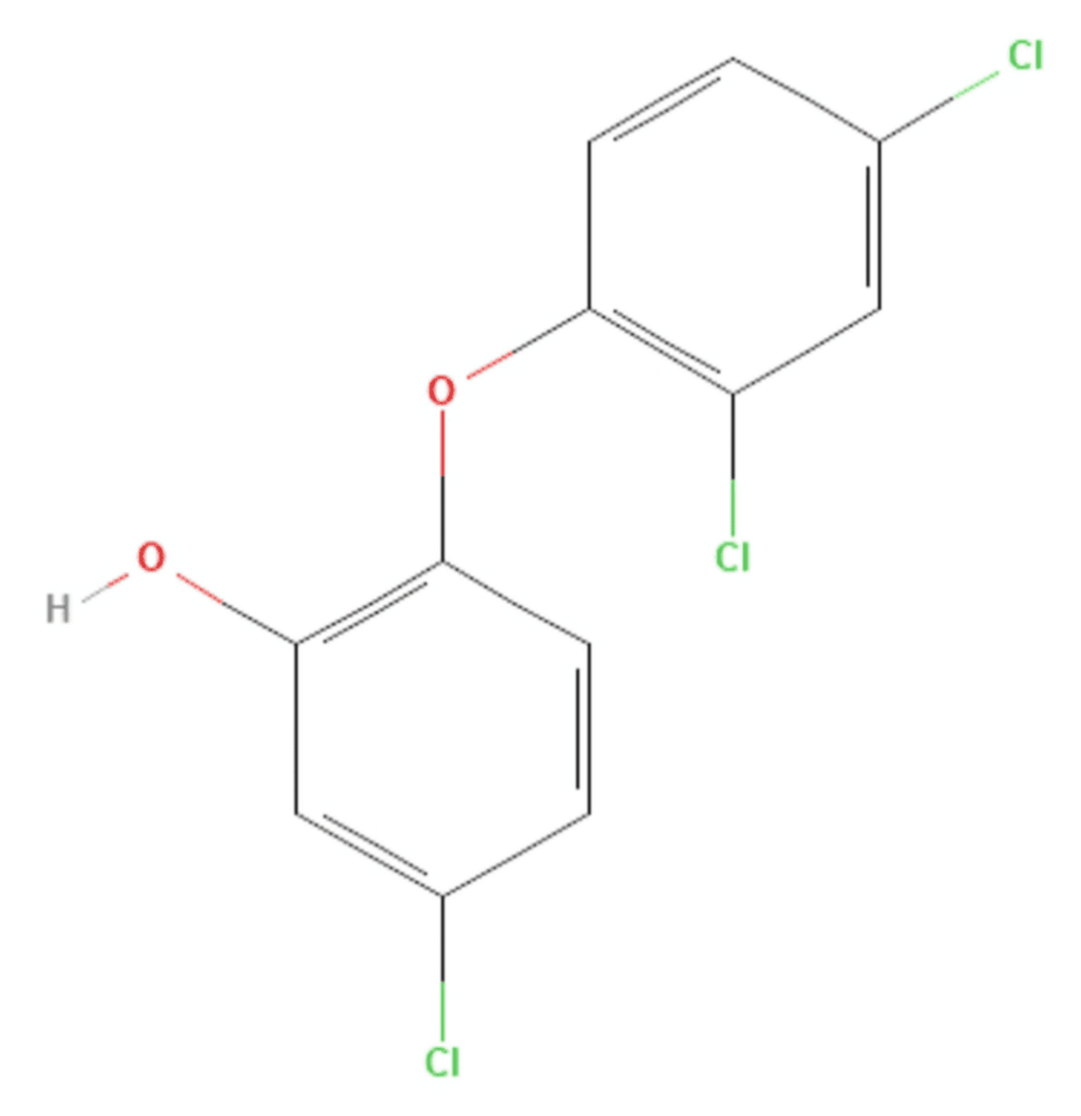
Chemical structure of triclosan O: oxygen; H: hydrogen; Cl: chlorine Reference: [10]

MITSU AB™ absorbable suture is a braided coated synthetic absorbable sterile poly(glycolide/L-lactide) surgical suture. It is composed of a copolymer made from 90% glycolide and 10% L-lactide. MITSU AB™ absorbable sutures are coated with a mixture containing equal parts of a copolymer of glycolide and lactide and calcium stearate, as well as the broad-spectrum antibacterial agent triclosan (2,4,4’-trichloro-2’-hydroxydiphenyl ether). The empirical formula of the copolymer is [(C2H2O2)(C3H4O2)]n, and calcium stearate is C36H70O4Ca. Poly(glycolide/L-lactide) copolymer and poly(glycolide/L-lactide) with calcium stearate exhibit non-pyrogenic properties (Figure 1).

MITSU AB™ absorbable sutures are available in undyed and dyed (D and C Violet No. 2) forms. Available in a broad range of suture sizes, 5-0 to 2 lengths, MITSU AB™ absorbable suture comes with standard stainless-steel needles of varying types and sizes. It complies with the Absorbable Surgical Suture requirements as per the United States Pharmacopeia (USP) and Sterile Synthetic Absorbable Braided Strands requirements as per the European Pharmacopoeia (EP).

Study outcome

The primary endpoints included the occurrence of superficial SSIs, monitored at discharge and at seven, 15, and 30 days post-surgery, in accordance with the Centers for Disease Control and Prevention (CDC) definitions. The presence of triclosan-resistant pathogens was determined using aseptic microbial cultures on Mueller Hinton agar infused with triclosan, where any microbial growth was interpreted as resistance. If resistance was observed, bacterial selection and microscopic characterization would be conducted. Additionally, wound healing was graded at each follow-up based on clinical appearance, with Grade A representing ideal healing without adverse reactions. Secondary endpoints included the measurement of postoperative incisional pain using the visual analogue scale (VAS) and the incidence of re-admissions or re-interventions due to SSI-related complications.

Statistical plan

Microsoft Excel (Microsoft Corp., Redmond, WA, USA) and IBM SPSS Statistics for Windows, V. 27.0 (IBM Corp., Armonk, NY, USA), were used for data cleaning and statistical analysis. Categorical and continuous variables were reported in proportions and mean ± SD.

Study approvals

Prior to the study, ethical committee approval was obtained from the Ojas Multispeciality Hospital Ethics Committee (approval number: ECR/1284/Inst/MH/2019/RR-24/NOV/2024) and the Dixit Hospital Institutional Ethics Committee (approval number: ECR/1599/Inst/GJ/2021/NOV/2024).

## Results

Demographics and baseline vitals

**Figure 2:**
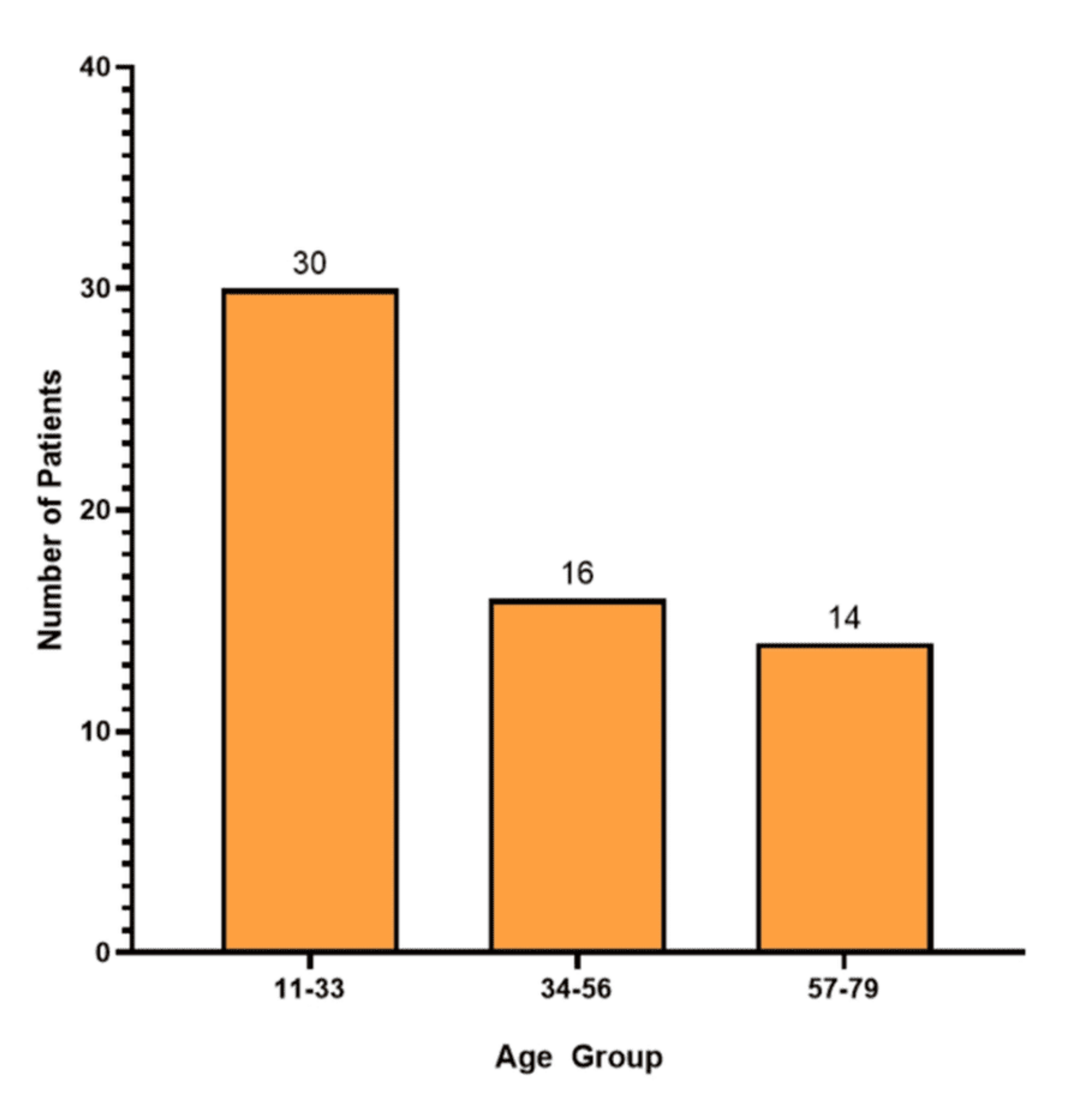
Age distribution

**Figure 3:**
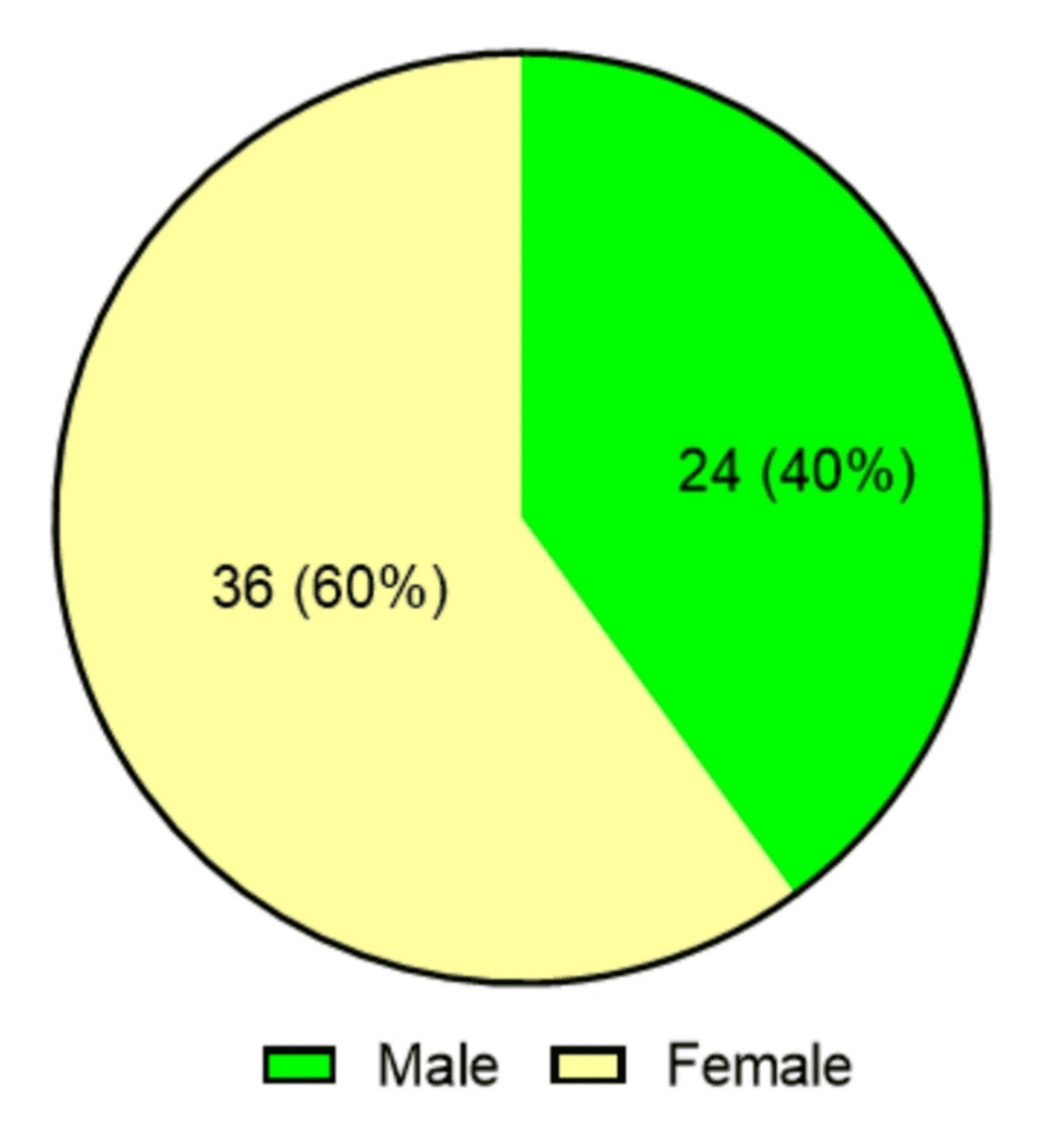
Gender distribution

The study cohort (n = 60) had a mean age of 38.5 ± 17.41 years, with half of the participants (50%) aged between 11 and 33 years (Figure 2). The sample demonstrated a female predominance (36 (60%) female; 24 (40%) male) (Figure 3). Anthropometric measurements indicated a mean height of 159.45 ± 9.48 cm, a weight of 61.12 ± 13.23 kg, and a BMI of 23.87 ± 4.43 kg/m². Comorbidity burden was low, with diabetes mellitus present in one patient (1.67%) and hypertension in five patients (8.33%) (Table 1).

**Table 1:** Demographics and baseline vitals

Variables	Frequency (%)
Age distribution (years)	
11-33	30 (50)
34-56	16 (26.67)
57-79	14 (23.33)
Mean age	38.50 ± 17.41
Gender	
Male	24 (40)
Female	36 (60)
Anthropometric data	
Height (cm)	159.45 ± 9.48
Weight (kg)	61.12 ± 13.23
BMI (kg/m²)	23.87 ± 4.43
Vitals	
Heart rate (bpm)	83.35 ± 9.23
Systolic blood pressure (mmHg)	127.63 ± 15.26
Diastolic blood pressure (mmHg)	82.63 ± 9.37
Temperature (°C)	37.11 ± 0.46
Blood oxygen (mmHg)	87.87 ± 10.07
Comorbidities	
Diabetes	1 (1.67)
Hypertension	5 (8.33)

Diagnosis and index procedure

Among the 60 patients assessed, the most common diagnosis was acute appendicitis (six cases; 10%), followed by lipomas at various anatomical sites (six cases; 10%) and laceration injuries, including the forehead, scalp, and extremities (12 cases; 20% combined). The most frequently performed procedure was suturing of lacerations (23 cases; 38.33%), followed by the excision of lipoma (six cases; 10%), episiotomy (four cases; 6.67%), and laparoscopic appendectomy (six cases; 10%) (Table 2).

**Table 2:** Diagnosis and index procedure

Variables	Frequency (%)
Diagnosis	
Acute appendicitis	6 (10)
Auricular hematoma	1 (1.67)
Carcinoma of the right breast	1 (1.67)
Cervical lymphadenopathy	1 (1.67)
Cervical mucus discharge with cervical opening	1 (1.67)
Chin laceration	2 (3.33)
Cholelithiasis	1 (1.67)
Closed laceration wound on the ring finger	1 (1.67)
Contused lacerated wound over the right index finger	1 (1.67)
Deep forehead laceration	1 (1.67)
Dermoid cyst on the back	1 (1.67)
Elbow trauma	1 (1.67)
Finger laceration	1 (1.67)
Anal fissure with Grade 2 internal hemorrhoids	1 (1.67)
Anal fissure with Grade 2 hemorrhoids	1 (1.67)
Forehead laceration	1 (1.67)
Foreskin trauma	1 (1.67)
Grade 3 internal hemorrhoids	1 (1.67)
Hand laceration	1 (1.67)
Iatrogenic right knee laceration	1 (1.67)
Inguinal hernia	1 (1.67)
Labor pain	1 (1.67)
Left breast fibroadenoma	1 (1.67)
Left knee trauma	1 (1.67)
Left thigh swelling	2 (3.33)
Leg laceration	1 (1.67)
Lipoma on the right toe	1 (1.67)
Lipoma on the back (cervical region C2)	1 (1.67)
Lipoma on the left leg (calf muscle)	2 (3.33)
Lower lip mucocele	1 (1.67)
Multinodular goiter	1 (1.67)
Paraphimosis	1 (1.67)
Perianal fistula	1 (1.67)
Peritonitis due to appendicular perforation	1 (1.67)
Right hand closed laceration wound	2 (3.33)
Right hand cellulitis	1 (1.67)
Right inguinal hernia	2 (3.33)
Right knee trauma	2 (3.33)
Right-sided torsion of the testis	1 (1.67)
Scalp laceration	2 (3.33)
Shoulder abscess	2 (3.33)
Superficial forehead laceration	2 (3.33)
Trauma on the hand	1 (1.67)
Traumatic chin laceration	1 (1.67)
Vaginal perforation	2 (3.33)
Index procedure	
Circumcision	2 (3.33)
Drainage and suturing	1 (1.67)
Episiotomy	4 (6.67)
Excision and drainage	1 (1.67)
Excision of fibroadenoma	1 (1.67)
Excision of lipoma	6 (10)
Excision of lymph nodes	1 (1.67)
Excision of mucocele	1 (1.67)
Excision of cyst	1 (1.67)
Excision of hematoma	1 (1.67)
Exploratory laparotomy	1 (1.67)
Fistulectomy	1 (1.67)
Hemorrhoidectomy	1 (1.67)
Hemorrhoidopexy with anal dilation	1 (1.67)
Laparoscopic appendectomy	6 (10)
Laparoscopic cholecystectomy	2 (3.33)
Laparoscopic transabdominal preperitoneal repair	2 (3.33)
Right-sided modified radical mastectomy	1 (1.67)
Scrotal exploration with right orchiectomy	1 (1.67)
Sphincterotomy with hemorrhoidectomy	1 (1.67)
Suturing of laceration	23 (38.33)
Total thyroidectomy	1 (1.67
Suture location	
Abdomen	1 (1.67)
Above the pubic bone	3 (5)
Antebrachium	1 (1.67)
Auricular lobe	1 (1.67)
Cubital joint	1 (1.67)
Distal phalanx	3 (5)
Femoral region	3 (5)
Finger extremities	1 (1.67)
Frontal forehead	1 (1.67)
Frontal region	4 (6.67)
Gastrocnemius region	1 (1.67)
Genus	1 (1.67)
Labia majora	2 (3.33)
Mentum region	2 (3.33)
Occipital region	2 (3.33)
Prepuce region	1 (1.67)
Radiocarpal joints	8 (13.33)
Rectum region	1 (1.67)
Scapular region	1 (1.67)
Scruff region	1 (1.67)
Strait region	1 (1.67)
Submandibular region	2 (3.33)
Temporal region	1 (1.67)
Thoracic region	1 (1.67)
Tibial region	1 (1.67)
Tunica albuginea	2 (3.33)
Upper outer quadrant	1 (1.67)
Vermillion border	12 (20)

Regarding suture locations, the abdomen was the most common site (12 cases; 20%), followed by the rectal region (eight cases; 13.33%) and gastrocnemius and femoral regions (four cases (6.67%) and three cases (5%), respectively) (Table 2).

Use device details

Among the 60 procedures, the most frequently used suture size was 3-0, employed in 50 cases (83.3%), and all sutures were absorbable. A round-bodied needle was used in 47 cases (78.3%), and the 40 cm bifurcation length was selected in the majority (55 cases; 91.7%). The mean number of stitches per procedure was 5.15 ± 1.98, with a mean wound closure time of 9.38 ± 3.67 minutes. In most patients, only one wound was treated using the study device (49 cases; 81.7%). Suture handling was rated as excellent (score 5) in 51 cases (85%) (Table 3).

**Table 3:** Use device details

Variables	Frequency (%)
Suture size	
2-0	10 (16.7)
3-0	50 (83.3)
Nature	
Absorbable	60 (100)
Type of needle	
Reverse	13 (21.7)
Round	47 (78.3)
Length of needle bifurcation	
20 cm	5 (8.3)
40 cm	55 (91.7)
Number of stitches (mean ± SD)	5.15 ± 1.98
Number of wounds treated with study device	
1	49 (81.67)
2	2 (3.33)
3	9 (15)
Time to wound closure (mean ± SD)	9.38 ± 3.67
Handling of suture	
4	9 (15)
5	51 (85)

Discharge and follow-up details

**Figure 4:**
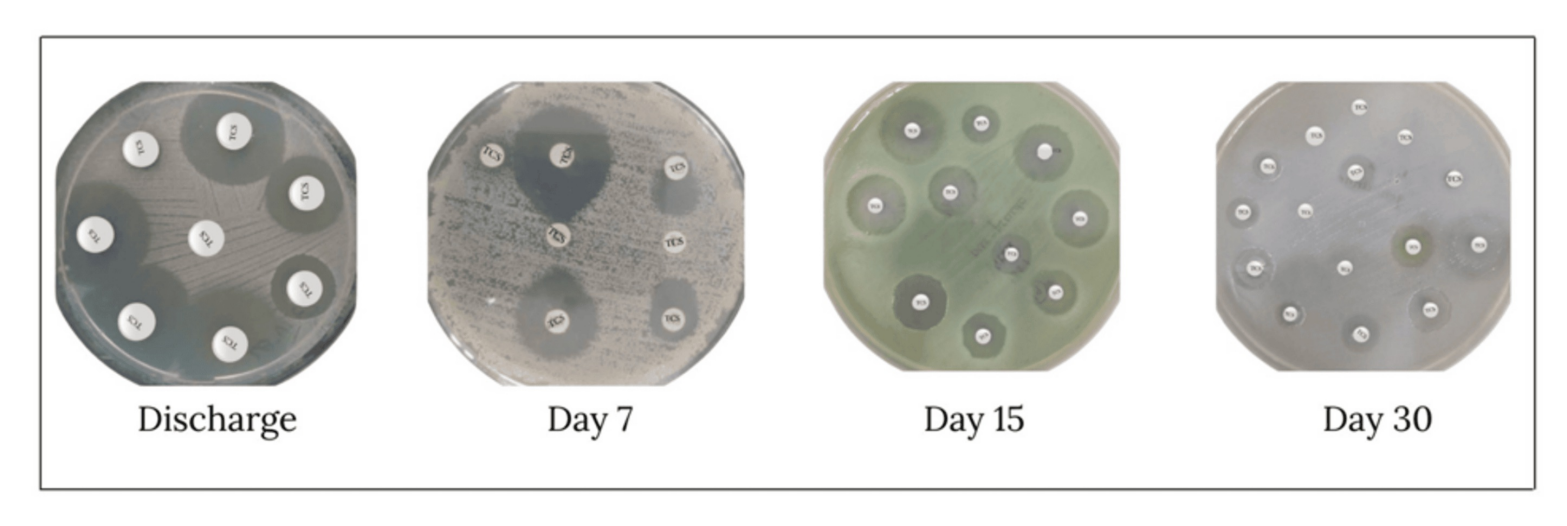
Zone of inhibition TCS: triclosan

All 60 patients (100%) achieved complete wound healing (Grade A) by day 30, with no reported adverse events, superficial SSIs, or need for re-admission or re-intervention across the study period. Microbial cultures remained negative, and no resistance to triclosan was detected at any time point. At the seven-day follow-up, 63.3% (38 patients) reported no pain, while 35% (21 patients) reported mild pain and only 1.7% (one patient) experienced moderate pain. By day 15, pain had resolved in 98.3% (59 patients), and by day 30, all patients (100%) were pain-free. The mean discharge time was 2.38 ± 1.61 days, and no serious adverse events (SAEs) were recorded (Figure 4, Table 4).

**Table 4:** Discharge and follow-up details AE/SAE: adverse event/serious adverse event; SSI: surgical site infection; VAS: visual analogue scale

Variables	Frequency (%)
Discharge time	
Length of stay (days)	2.38 ± 1.61
AE/SAE	0 (0)
Superficial SSI	0 (0)
Aseptic microbial culture performed	60 (100)
Resistance to triclosan	0 (0)
7-day follow-up	
Adverse event	0 (0)
Aseptic microbial culture performed	60 (100)
Resistant to triclosan	0 (0)
VAS score	
Mild	21 (35)
Moderate	1 (1.67)
No pain	38 (63.33)
Superficial SSI	0 (0)
Re-admission due to SSI-related complications	0 (0)
Re-intervention due to SSI-related complications	0 (0)
Wound healing grade	
Grade A	60 (100)
15-day follow-up	
Adverse event	0 (0)
Aseptic microbial culture performed	60 (100)
Resistant to triclosan	0 (0)
VAS score	
Mild	1 (1.67)
No pain	59 (98.33)
Superficial SSI	0 (0)
Re-admission due to SSI-related complications	0 (0)
Re-intervention due to SSI-related complications	0 (0)
Wound healing grade	
Grade A	60 (100)
30-day follow-up	
Adverse event	0 (0)
Aseptic microbial culture performed	60 (100)
Resistant to triclosan	0 (0)
VAS score	
No pain	60 (100)
Superficial SSI	0 (0)
Re-admission due to SSI-related complications	0 (0)
Re-intervention due to SSI-related complications	0 (0)
Wound healing grade	
Grade A	60 (100)

## Discussion

MITSU AB™ absorbable triclosan-coated sutures demonstrated excellent clinical outcomes in a real-world surgical setting. The complete absence of superficial SSIs, triclosan-resistant microbial colonization, and adverse events across 60 diverse surgical cases underscores the safety and efficacy of this suture material.

Our findings are consistent with prior clinical and experimental research showing that triclosan-coated sutures significantly reduce the risk of early microbial colonization and SSIs. Studies involving Vicryl Plus and Monocryl Plus have demonstrated similar antimicrobial effects by creating a localized zone of inhibition that is particularly effective during the early postoperative period when the wound is most vulnerable [6]. In our study, MITSU AB™ maintained this expected performance profile with full wound healing observed in all patients and no emergence of triclosan resistance, an important outcome given the growing concerns about antimicrobial resistance.

Triclosan acts by disrupting bacterial fatty acid synthesis, particularly targeting the enoyl-acyl carrier protein reductase (ENR) enzyme, a mechanism that provides broad-spectrum activity against Gram-positive and Gram-negative bacteria [11]. However, resistance can occur through multiple mechanisms such as efflux pump activation, target site mutations, or biofilm formation especially in organisms like *Pseudomonas aeruginosa* and *Klebsiella pneumoniae* [12]. Our surveillance detected no such resistance, possibly due to the short duration of exposure to triclosan and the controlled clinical environment.

The robust wound healing outcomes (Grade A in all cases) also support the hypothesis that triclosan-coated sutures not only reduce infection but may promote optimal healing conditions by lowering local microbial load and inflammation. Inflammatory modulation has been suggested as a secondary benefit of antimicrobial-coated sutures, potentially resulting in better tissue integration and reduced scarring [13].

Furthermore, the high suture handling score (85% rated as excellent) and consistent usage patterns across varied anatomical locations reinforce the usability of MITSU AB™ in clinical practice. Ease of handling, minimal tissue drag, and predictable knot security are essential features for surgical efficacy, especially in emergency or resource-limited settings.

However, it is also important to recognize that while our findings are promising, long-term data and larger sample sizes are needed to definitively rule out the possibility of triclosan resistance under broader conditions. Some studies have reported limited efficacy of triclosan in preventing SSIs in clean-contaminated or high-risk wounds, suggesting that the protective benefit may vary with procedure type and microbial exposure. [14] Moreover, the lack of comparator arms in this single-arm design limits the ability to directly assess relative benefit over non-coated sutures.

Lastly, given global efforts to combat antimicrobial resistance, surveillance for triclosan resistance remains vital. The balance between infection prevention and resistance development should be continually reassessed, especially as triclosan remains prevalent in both healthcare and consumer environments [15].

Limitation

The absence of a comparator group (non-coated sutures) limits the ability to assess the relative effectiveness of triclosan coating in reducing SSIs or resistance emergence. Although 60 patients were evaluated, the sample is relatively small for generalizing findings across diverse surgical populations, particularly for rare adverse events or resistance emergence. A 30-day follow-up may not be sufficient to detect late-onset infections or delayed microbial resistance patterns, especially in patients with comorbidities or impaired immunity. One of the limitations of this study is the inclusion of a heterogeneous mix of surgical procedures encompassing clean, clean-contaminated, and contaminated cases.

Strength

Conducted in routine clinical practice, the study reflects real-life surgical workflows and patient responses, enhancing external validity. Multiple follow-up points and standardized assessment of wound healing, pain, and microbial colonization offer a robust data collection framework. The consistently high handling scores and practical utility across various surgical procedures highlight the usability and versatility of MITSU AB™ sutures.

## Conclusions

The results of this post-marketing surveillance study suggest that MITSU AB™ absorbable triclosan-coated sutures are safe, effective, and user-friendly across a range of surgical scenarios. With complete wound healing, absence of SSIs, and no detectable triclosan resistance, the sutures offer a viable option for infection prevention during the critical postoperative period. Although concerns about antimicrobial resistance remain globally relevant, this study supports the continued use of triclosan-coated sutures under monitored and judicious conditions. Future randomized studies with larger cohorts and longer follow-ups are warranted to strengthen the evidence base and monitor for emerging resistance trends.
